# A protocol for the delivery of cannabidiol (CBD) and combined CBD and ∆^9^-tetrahydrocannabinol (THC) by vaporisation

**DOI:** 10.1186/2050-6511-15-58

**Published:** 2014-10-16

**Authors:** Nadia Solowij, Samantha J Broyd, Hendrika H van Hell, Arno Hazekamp

**Affiliations:** 1 School of Psychology, Ψ-P3: Centre for Psychophysics, Psychophysiology and Psychopharmacology and Illawarra Health and Medical Research Institute, University of Wollongong, Wollongong, NSW 2522, Australia; 2 Department of Plant Metabolomics, Faculty of Science, Leiden University, Einsteinweg 55, 2333 CC Leiden, The Netherlands

**Keywords:** Cannabinoids, Cannabidiol (CBD), ∆^9^-Tetrahydrocannabinol (THC), Vaporisation, Intrapulmonary Administration

## Abstract

**Background:**

Significant interest has emerged in the therapeutic and interactive effects of different cannabinoids. Cannabidiol (CBD) has been shown to have anxiolytic and antipsychotic effects with high doses administered orally. We report a series of studies conducted to determine the vaporisation efficiency of high doses of CBD, alone and in combination with ∆^9^-tetrahydrocannabinol (THC), to achieve faster onset effects in experimental and clinical trials and emulate smoked cannabis.

**Methods:**

Purified THC and CBD (40 mg/ml and 100 mg/ml respectively) were loaded onto a liquid absorbing pad in a Volcano® vaporiser, vaporised and the vapours quantitatively analysed. Preliminary studies determined 200 mg CBD to be the highest dose effectively vaporised at 230°C, yielding an availability of approximately 40% in the vapour phase. Six confirmatory studies examined the quantity of each compound delivered when 200 mg or 4 mg CBD was loaded together with 8 mg of THC.

**Results:**

THC showed 55% availability when vaporised alone or with low dose CBD, while large variation in the availability of high dose CBD impacted upon the availability of THC when co-administered, with each compound affecting the vaporisation efficiency of the other in a dynamic and dose-dependent manner. We describe optimised protocols that enable delivery of 160 mg CBD through vaporisation.

**Conclusions:**

While THC administration by vaporisation is increasingly adopted in experimental studies, often with oral predosing with CBD to examine interactive effects, no studies to date have reported the administration of CBD by vaporisation. We report the detailed methodology aimed at optimising the efficiency of delivery of therapeutic doses of CBD, alone and in combination with THC, by vaporisation. These protocols provide a technical advance that may inform methodology for clinical trials in humans, especially for examining interactions between THC and CBD and for therapeutic applications of CBD.

**Trial registration:**

Current Controlled Trials ISRCTN24109245

## Background

Scientific interest in understanding the effects of cannabinoids in humans has grown in recent years with the recognition that different compounds within cannabis plant matter can have vastly different but also synergistic effects. The primary psychoactive constituent of cannabis, ∆^9^-tetrahydrocannabinol (THC), has therapeutic effects in humans, but has also largely been associated with a range of adverse effects, including the induction of psychotic-like symptoms and memory impairment [1]. Another major constituent of cannabis plant matter, cannabidiol (CBD), has been shown to have anxiolytic and antipsychotic properties [2,3] and to ameliorate some of the adverse effects of THC [4,5]. THC and CBD have been shown to have opposing effects on regional brain activation in a range of cognitive tasks [6]. Studies of administration of these compounds to humans have relied on the oral administration of CBD and either smoked, vaporised, oral or intravenous administration of THC. Oromucosal sprays containing THC and CBD in a 1:1 ratio (nabiximols (Sativex^R^)) are increasingly being studied for therapeutic efficacy, but these deliver low doses, and higher doses of CBD in particular are thought to be required for meaningful modification of clinical outcomes [7]. Orally administered cannabinoids result in slow and erratic absorption with limited and highly variable bioavailability [8,9]. Smoking and intravenous administration produce reliable and similar pharmacokinetic profiles [9,10], but respectively carry toxic risks and loss of active drug by combustion, or are invasive.

Intrapulmonary administration of cannabinoids is regarded as an effective mode of delivery since it results in fast onset of action and high systemic bioavailability [9]. The vaporisation of cannabinoids (heating plant matter or pure compounds to a temperature where active cannabinoid vapours form but below the point of combustion) is a safe method of intrapulmonary administration because it avoids risks associated with smoking and the formation of pyrolytic toxic compounds as a result of combustion [11]. While some studies have reported on the effects of vaporised THC e.g. [12-14], no study has yet reported on CBD administered via vaporiser. Oral dosing with CBD impedes the potential to examine its interactive effects with THC when the two compounds are administered simultaneously, a scenario with ecological validity for understanding effects in recreational and medicinal cannabis users. Alternative routes of administration of CBD with rapid action, such as vaporisation, would also benefit further investigations of its therapeutic potential as an anxiolytic or antipsychotic, among other applications.

The purpose of this article is to describe methodology developed for the administration of THC alone, CBD alone and their co-administration by means of a vaporiser for studies of acute cannabinoid effects in humans. Since the therapeutic (anxiolytic and antipsychotic) doses of orally administered CBD have generally been quite high (e.g. around 600 mg), we attempted to determine whether it may be possible to vaporise similarly high doses of CBD both alone, and in combination with THC. We report optimised protocols for the delivery of 160 mg or more of CBD by vaporisation.

## Methods

Purified THC (purity >98%) was produced and quantified as reported previously [15,16]. Purified CBD (purity >98%) was purchased from Echo Pharmaceuticals (Weesp, The Netherlands). The cannabinoids were stored as ethanolic solutions at −20°C at a concentration of 40 mg/ml (THC) and 100 mg/ml (CBD). All organic solvents were HPLC or analytical grade and were purchased from JT Baker (Deventer, The Netherlands). Glass fibre filters (Cambridge type, borosilicate glass, 92 mm diameter) and tightly fitting filter holders for capturing the vapour were obtained from Borgwaldt Technik GmbH (Hamburg, Germany).

The Volcano® vaporiser device (type Volcano® ‘Digit’) was obtained from Storz & Bickel GmbH & Co. (Tuttlingen, Germany) and was used according to the manual as provided by the manufacturer. A standard volume balloon (length of 60 cm, volume around 10 litres) was used for all experiments unless specified otherwise. The temperature was set to 230°C, unless specified otherwise. All experiments were carried out in a standard laboratory fume hood under constant ventilation with an ambient room temperature of about 20°C and a humidity of 40–60%.

Crystalline form CBD (preliminary experiments) or ethanolic solutions of CBD and THC (confirmatory and replication studies) were loaded onto the vaporiser filling chamber via a liquid pad – a removable disc made of tightly packed stainless steel wire mesh – as supplied by the manufacturer of the Volcano®. For the confirmatory and replication studies, the desired dose of cannabinoids (see Table 1) was applied in aliquots of maximum 400 μl, in order to prevent overloading of the liquid pad, which may result in leaking. After each application, ethanol was evaporated using the Volcano® vaporiser set at 100°C for 30 seconds. THC and CBD (with boiling points well over 160°C) were not affected by this treatment, and remained as pure compounds on the liquid pad. Before each new experiment the filling chamber was thoroughly cleaned with ethanol and allowed to dry at ambient temperature. A new liquid pad was used for each experiment.

**Table 1:** Cannabinoid doses applied and corresponding volume of ethanolic solutions (of 40 mg/ml THC or 100 mg/ml CBD stock solutions) for each of 6 experiments

	**THC (mg)**	**volume (μl)**	**CBD (mg)**	**volume (μl)**
Experiment 1	8	200	-	-
Experiment 2	-	-	4	40
Experiment 3	8	200	4	40
Experiment 4	-	-	200	5x400
Experiment 5	4	100	200	5x400
Experiment 6	8	200	200	5x400

For each experiment, the filling chamber fitted with a loaded liquid pad was placed onto the vaporiser stabilised at the desired temperature. The balloon was then immediately attached and the ventilation was started. When the balloon was completely inflated, ventilation was stopped and the content of the balloon was processed for analysis within 2 min.

### Extraction of cannabinoids from the vapour and the liquid pad

Cannabinoids were recovered from the balloon by slowly aspiring the vapour through a glass-fibre filter designed to capture particles >0.1 microns with the use of a membrane pump. Cannabinoids were then extracted from the filter by mechanically shaking the filter for 10 minutes with 40 ml of ethanol in 50 ml serum tubes. Supernatant was transferred to a 100 ml volumetric flask. The procedure was repeated twice more with 25 ml of ethanol, solutions were combined and the total volume was brought to 100 ml with ethanol. Finally the solution was filtered through a 0.45 μm PTFE syringe filter, and stored at −20°C until analysis by HPLC.

A previous study showed that cannabinoids present in the vapour are trapped >95% onto the glass fibre filter [11]. For the present study, recovery was determined by spiking filters in triplicate with THC (8 mg) or CBD (200 mg) solution, allowing the filters to dry, and performing the extraction procedure described above. Recovery from the filter was found to be 99.3 ± 1.1% for THC and 99.1 ± 0.6% for CBD.

### HPLC analysis of vapour extracts

An Agilent 1200 series HPLC was used for quantitative analysis of the vapour extracts. The system consisted of a G1322A degasser, G1311A quaternary pump, G1367B automated liquid sampler, and G1315D diode-array (DAD) detector (Agilent Technologies Inc.). The software used was Chemstation Rev. B03.02.

The analytical column was a Phenomenex C18 UHPLC column, type Kinetex (3.0×100 mm, 2.6 μm). The mobile phase consisted of a mixture of methanol–water (both containing 25 mM of formic acid) in gradient mode; the proportion of methanol was linearly increased from 75 to 100% over 10 minutes, and then kept constant for 1 minute. The column was re-equilibrated under initial conditions for 4 min. Flow-rate was 0.5 ml/min and injection volume was 2 μl. Eluted compounds were detected at a wavelength of 228 nm. All determinations were carried out at a column temperature of 40°C. The HPLC method used was previously described ([11]; adapted in [17]) and validated again here.

The method was validated for linearity, accuracy, precision, and limit of quantification (LOQ). To establish the linearity of the method, 6-point calibration curves were prepared for THC and for CBD in the range of 0.01 to 1.0 mg/ml, with each analyte concentration analysed in 3-fold. Repeatability was determined by analysing six replicates of the same sample (THC 1 mg/ml). Intraday precision was calculated by analysing all analyte samples three different times in a day. The same procedure was followed for four different days to determine interday precision. Recovery was determined by spiking glass-fiber filters in triplicate with THC (8 mg) or CBD (200 mg) solution, allowing the filters to dry, and performing the extraction procedure described above.

The method showed excellent validation results, with an average linearity of 0.9996 and 0.9994 for THC and CBD, respectively. Intraday precision was 2.0% (THC) and 1.8% (CBD), while interday precision was 4.1% (THC) and 4.9% (CBD). Limit of quantification (THC and CBD) was set equal to the lowest analyte concentration analysed, which was 0.01 mg/ml. Recovery from the filter was found to be 99.3 ± 1.1% for THC and 99.1 ± 0.6% for CBD.

### Preliminary experiments

A series of preliminary experiments was conducted first to determine optimum forms of CBD for vaporisation (crystalline versus ethanolic solution), maximum volumes of ethanolic solution that could be held by the liquid pad, and hence maximum doses that could be achieved. The quantity of cannabinoids delivered into each balloon and the residue remaining on the liquid pad were quantified by HPLC. Solubility concerns for CBD in ethanolic solution and anticipated inability of the liquid pad to hold the large quantities of liquid, led to preliminary testing using CBD in crystalline form. 100 mg CBD was directly loaded onto the liquid pad and vaporised at two different temperatures, 210°C and 230°C, into three subsequent balloons (to capture as much CBD vapour as possible) [Pilot study 1; see Additional file 1]. Next, higher doses of crystalline CBD – 200 mg and 300 mg – were attempted to be vaporised into a single standard balloon at 230°C [Pilot study 2; see Additional file 1]. In Pilot study 3 [see Additional file 1], 200 mg of crystalline CBD was vaporised at 230°C into standard size and extra large (XL; 90 cm) size balloons to determine whether the amount delivered could be increased by allowing a longer time for compounds to vaporise. Finally, we proceeded to establish whether CBD in ethanolic solution of similar concentration would vaporise similarly to what we had established for crystalline CBD [Pilot study 4; see Additional file 1]. This was deemed to be particularly pertinent since pure THC is generally vaporised dissolved in ethanol and it would be desirable for randomised administration studies of both compounds to humans to deliver each compound similarly, and enable ethanol to be used in a placebo condition. Due to the resinous, sticky nature of CBD, we started with 100 mg CBD dissolved in a 10% ethanolic solution loaded onto the liquid pad of the vaporiser by pipetting 1 ml of solution, in several separate aliquots at a time in order to prevent overloading of the liquid pad and vaporising ethanol each time at 100°C for 30 sec. A further aim for Pilot study 4 was to assess the potential for co-administration of THC and CBD through the vaporiser. 10 mg THC (0.4 ml from a 4% ethanolic solution) was vaporised alone and in combination with the 100 mg CBD, into a normal size balloon at 230°C. This series of preliminary studies was used to determine the optimum dose delivery and vaporisation protocols for planned use in a randomised controlled trial (RCT) of cannabinoid (THC and CBD) administration to humans [18]. Final confirmatory and replication studies were undertaken using doses described in Table 1 as 6 separate experiments repeated in triplicate, with results described below.

### Confirmatory/replication studies

Having identified a range of properties pertinent to optimising vaporisation of CBD, we then selected specific doses for confirmation and replication toward the planned RCT. A dose of 8 mg THC was selected as the desired dose to administer based on previous studies of THC administration to humans through a vaporiser e.g. [12-14]. This was designed to achieve clinically relevant intoxication without significant adverse effects. For CBD, the goal of the planned RCT was to deliver a dose close to, or equivalent to, the 600 mg CBD doses that have been demonstrated to have psychoactive (regional brain activation) or therapeutic effects in humans, when administered orally e.g. [3,6]. Since our preliminary experiments indicated that more than 200 mg CBD could not be vaporised efficiently into a single balloon, we decided to administer two balloons with a 200 mg loaded dose of CBD for vaporisation into each to achieve a higher administered dose. A further goal of the RCT was to administer THC and CBD in combination, at high and low doses of CBD (keeping the dose of THC constant). The low dose of CBD was set at 4 mg, to emulate a 2:1 THC:CBD ratio that had been more common in street level cannabis products (although highly variable) [19,20]. Thus, a series of 6 experiments tested the quantity of THC and/or CBD delivered into each balloon when THC and CBD were each vaporised alone, and in combination.

The temperature setting of the Volcano® vaporiser for each experiment was kept constant at 230°C. The liquid pad was loaded with CBD and/or THC, each in ethanolic solution according to the doses for each experiment as outlined in Table 1. All experiments used a normal size balloon and each experiment was performed in triplicate (i.e. 6 experiments repeated three times) using a fresh dose, a new liquid pad and a new balloon each time, and a single vaporisation to fill the balloon.

## Results

### Preliminary experiments

Results from the series of pilot studies [see Additional file 1] determined that vaporisation of CBD at 230°C was superior to 210°C, in terms of the quantity of CBD delivered into the balloon (Pilot study 1). A saturation effect appeared to occur at 200 mg CBD, with greater quantities resulting only in excessive resinous residue remaining on the liquid pad (Pilot study 2). A greater amount of CBD vapour was delivered into the XL balloon than into the standard size balloon (Pilot study 3); the amount delivered into the standard balloon was similar to that of the previous pilot study, thus replicating the delivered dose of approximately 25% following the loading and vaporising of 200 mg CBD. A decision was made to continue experiments with the normal size balloon for its greater ease (shorter duration) of inhalation (for the participants in the planned RCT [18]). 100 mg of CBD dissolved in ethanol (Pilot study 4) delivered a similar amount of vapour as was observed in the prior work using 100 mg solid form CBD, about 25% of the loaded dose, thus replicating this finding and establishing the viability of loading CBD dissolved in ethanolic solution. The amount of vaporised CBD delivered into the balloon was approximately half that observed with 200 mg CBD loaded (the proportion being delivered as vapour remaining the same – approximately 25%). Finally, this study also determined that the presence of CBD (100 mg) decreased the amount of THC vaporised into the standard balloon after loading 10 mg THC, by about half, with a small reduction also to the delivered dose of CBD [see Additional file 1 for depiction of data from each Pilot study].

### Confirmatory/replication studies

**Figure 1:**
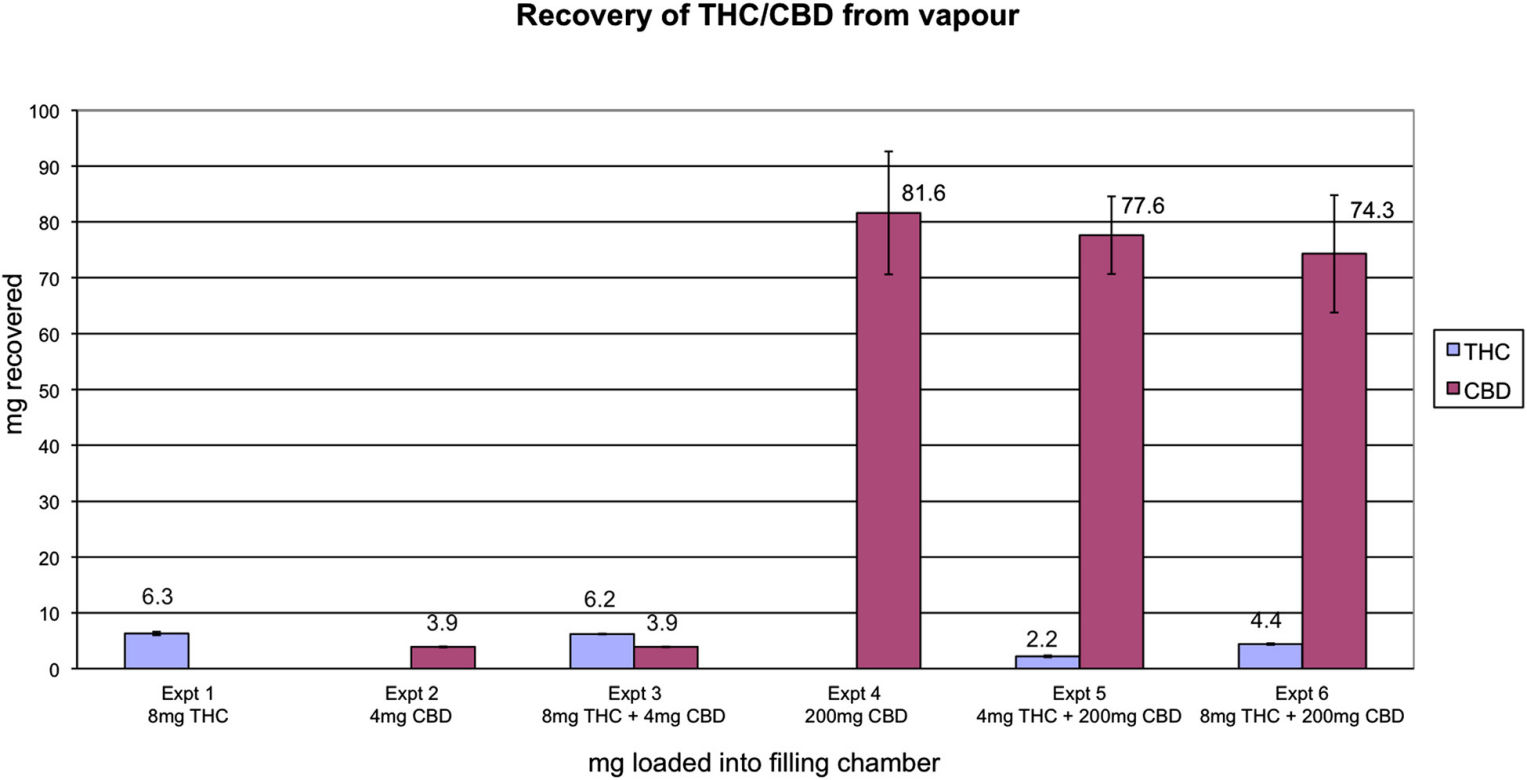
**HPLC quantification of THC and CBD in vapour delivered into a 60 cm (approximately 10 litre) balloon after vaporisation of 8 mg THC, and 4 mg CBD or 200 mg CBD, loaded alone and in combination onto the liquid pad of a Volcano® vaporiser and vaporised at 230°C.** Data represent averages across three repetitions; error bars are SEM.

Results from the series of 6 experiments are depicted in Figure 1. Each column shows the average amount of THC or CBD (mg, SEM), HPLC quantified from the vapour delivered into the balloon from three repetitions.Experiment 1 indicated that 8 mg THC when vaporised alone delivered approximately 6.3 (SD 0.53) mg THC into the balloon for inhalation. Experiment 2 indicated that 4 mg CBD when vaporised delivered approximately 3.9 (SD 0.13) mg CBD into the balloon. When 4 mg CBD was combined with 8 mg THC (Experiment 3), there was no significant change to the amount of each compound delivered from when each was delivered alone (THC: p > 0.96; CBD: p > 0.65). Experiment 4 determined that when 200 mg CBD was vaporised, almost 82 mg CBD (41%) was delivered into the balloon on average, but there was significant variability (large standard errors). In Experiment 6, the combination of 8 mg THC with 200 mg CBD when vaporised, resulted in reduced delivery of each compound into the balloon relative to when each was vaporised alone: 4.4 (SD 0.22) mg THC was delivered (55%) (significantly different from Experiment 1, p < 0.05; and Experiment 3, p < 0.015), and 74.3 (SD 18.17) mg CBD was delivered (37.2%) (but this did not differ statistically from Experiment 4, p > 0.79); again there was large variation for CBD. Experiment 5 was subsequently conducted to establish what would happen if half the quantity of THC (4 mg) were combined with 200 mg CBD. This was performed in order to determine whether a second balloon could be administered to participants with additional THC to equalise the quantity of THC delivered when in combination with high dose CBD, to that when administered alone. Again the quantity of THC delivered into the balloon was approximately halved: 2.2 (SD 0.28) mg (55%), and slightly more CBD was delivered: 77.6 (SD 12.06) mg (38.9%). Experiments 5 and 6 indicated that a saturation effect in the vapour may occur when the two compounds are vaporised together at high CBD doses (but not at low CBD doses as in Experiment 3). Despite the trend evident in Figure 1, the quantity of CBD delivered across experiments 4, 5 and 6 did not differ statistically (p > 0.94).

## Discussion

To our knowledge, this is the first reported methodological study toward intrapulmonary administration of CBD alone and in combination with THC in the literature. We provide here a systematically optimised protocol for the delivery of CBD, and CBD together with THC, by means of vaporisation. Vaporisation provides a safe and efficient delivery system for cannabis and cannabinoid compounds, avoiding the respiratory toxins inherent in smoking, making it useful for clinical trials. The rapid delivery into the bloodstream producing immediate effects made possible by this method of administration may benefit in particular human research studies that have to date relied on oral administration of CBD, with its delayed onset of effects and inherent problems regarding bioavailability and liver metabolism. Benefits may also extend to research toward further development of CBD for therapeutic use for a range of indications and disorders including amongst others, schizophrenia [3] and anxiety disorders [21]. Administration of THC and CBD together mimics the natural constituency of cannabis plant matter, prior to the more recent selective breeding that has resulted in the reduction or even elimination of CBD while maximising THC content [20], and the methods described here can assist with enabling researchers to examine the effects of the two compounds delivered simultaneously. The ability to do this by vaporisation overrides the necessity to pre-dose with oral CBD hours prior to the administration of inhaled THC, which in itself results in altered pharmacokinetics and pharmacodynamics [22] (but see also [23,24]), and provides for the development of a better understanding of the interaction between the two compounds, enabling well-designed experiments of dose-dependent effects.

The experiments performed in triplicate here determined the doses that could be achieved in our own RCT as follows (to demonstrate the application of the protocols we report here) [18]. Five conditions were deemed to be achievable and to ensure blinding to drug condition and enable comparability of conditions, by administering two normal size balloons to each participant on each occasion, using ethanolic solutions of 4% THC and 10% CBD: 1) 8 mg THC alone (200 μl in first balloon plus 200 μl pure ethanol in second balloon); 2) 400 mg CBD alone (2 ml in each balloon); 3) 8 mg THC + 4 mg CBD (200 μl and 40 μl respectively in first balloon, 200 μl pure ethanol in second balloon); 4) 12 mg THC + 400 mg CBD (200 μl and 2 ml respectively in first balloon, 100 μl and 2 ml respectively in second balloon); 5) pure ethanol as placebo (200 μl in each balloon). Where only ethanol was administered, the ethanol vapours were blown off as described above, such that only mildly ethanol vapour flavoured air was delivered. Condition 3 aimed to examine the effects of a low dose of CBD when combined with THC, emulating the effects of ‘street level’ cannabis (as it once was), and for comparative purposes with the high dose CBD with THC condition (Condition 4), the latter emulating the previously determined therapeutic effects of high doses of oral CBD (described above, here with maximum feasible dose achievable through vaporisation). The results of the experiments reported here determined that the quantities of cannabinoids actually delivered via vaporisation would be comparable across the primary conditions of interest: the two balloon and adjusted THC dose method delivered approximately 6.2-6.6 mg THC across each of the conditions with THC (conditions 1, 3 and 4), whilst acknowledging the variability for CBD in delivering approximately 150–160 mg CBD across the two balloons in the two high dose CBD conditions (2 and 4). The results also established the almost 100% dose delivered for the low (4 mg) CBD dose when administered in combination with THC (Condition 3).

A limitation of this methodological report is that it has focused entirely on the technical aspects of feasibility of vaporisation of CBD (alone and in combination with THC). While these experiments have determined the amount of drug that can be loaded on the liquid pad of the Volcano® vaporiser and the proportion actually delivered into the balloon as vapour, further research is required to take this to the next step of determining the amount actually taken up by human inhalers of the vapours, how much is exhaled following controlled breath holding protocols, and the bioavailability of these compounds when administered through a vaporiser, as has thus far been determined for THC only [11,12]. The work reported here is the necessary first step for any further studies of this type with human subjects. Further limitations to consider with the vaporisation route of administration are that despite attempts to adhere to controlled breath holding, there may be variability in absorption related to the depth of respiration, individual patterns of breathing and coughing, as well as the variable size of particles in the vapour adhering at differing points within the respiratory system (e.g. in the throat) versus reaching the alveoli where absorption is most rapid and efficient.

The results from the series of studies conducted indicate that with small doses of CBD, such as the 4 mg we applied, 97.5% may be delivered into the balloon following vaporisation. With THC (8 mg) up to 80% of the dose loaded was delivered into the balloon, substantially greater than the 54% previously reported [11], possibly due to improvements in the heating efficiency of the vaporiser. With high dose CBD, only about 40% of the dose was delivered, indicating that evaporation does not increase linearly with the loaded dose. An optimal dose of 200 mg CBD can be effectively and efficiently vaporised to deliver approximately 80 mg of CBD. Human research participants may be asked to inhale two normal size balloons (standard with the Volcano® vaporiser) to achieve a combined dose of approximately 160 mg. A larger dose may be delivered if the CBD is vaporised into larger (XL) balloons, but these are 1.5 times larger than the normal size balloons, requiring significantly more inhalations, while the resultant dose delivered is not 1.5 times greater. The presence of both compounds reduces the delivery of each when the dose of CBD is high, likely due to saturation effects in the vapour. Further adjustments could potentially be made to vaporisation protocols and the doses loaded to try to better achieve the desired dose delivered. For example, we opted to have participants inhale two standard size balloons to maximise the dose of CBD delivered, judging it to be impracticable to have participants inhale two XL size balloons (because of their large volume and the time restraints and other demands on participants in our RCT); for other experiments researchers may opt to use one or two XL size balloons and achieve greater dosing by this means, with less wastage of the CBD dose loaded. Further, using our data reported above, in order to compare the effects of loaded doses of 8 mg THC and 400 mg CBD in combination, with the effects of each compound separately, we opted to load the vaporiser with 8 mg THC and 200 mg CBD for delivery into the first balloon (vaporised to deliver approximately 4.4 mg THC and 74 mg CBD), and we loaded 4 mg THC and 200 mg CBD for delivery into the second balloon (vaporised to deliver 2.2 mg THC and 78 mg CBD). The total doses delivered from these two balloons equal 6.6 mg THC and 152 mg CBD; this achieved equivalence broadly with the 6.2 mg THC and 163 mg CBD delivered when each compound was delivered separately. Alternatively, THC and CBD could be loaded and vaporised separately into two balloons that the participant inhales sequentially, but this could result in quite different pharmacokinetic and pharmacodynamic effects compared to simultaneous administration of the two compounds and the methodology depends upon the questions to be addressed in the research.

## Conclusions

Many questions arise regarding additive, synergistic and interactive effects of THC and CBD and the field is ripe for the further investigation of these intriguing compounds. The methods, protocols and preliminary data presented here established the optimal efficiency of delivery of both low and high doses of CBD, alone and in combination with THC, by vaporisation. While intrapulmonary administration generally results in high systemic bioavailability, the extent of absorption of the high doses of CBD that we were able to achieve, relative to oral doses of 600 mg, remains to be determined, as do their potential therapeutic effects. The studies informed the development of methods for a randomised controlled trial of simultaneous acute administration of these cannabinoids by vaporisation to human research participants in our laboratory [18], and may assist researchers with designing their own future clinical trials and experimental human studies^a^.

## Endnote

^a^A note of caution to future researchers. CBD when vaporised produces dense vapour that is irritating to the throat for some participants and generates sometimes significant coughing. We have facilitated the comfort of participants inhaling CBD vapours by offering small sips of water or juice, ice or sweets to suck and soothe the irritation, or sometimes unmedicated cough lozenges. Further, the dense vapours produced by CBD are visibly different to the less dense vapours produced by THC. It is recommended for blinding purposes to mask these differences by covering the balloon (e.g. with an opaque plastic bag or fabric cover).

## Competing interests

All authors declare that they have no competing interests. AH is Head of Research and Development at Bedrocan BV, The Netherlands, who had no further role in this study.

## Authors’ contributions

NS conceived of the study, designed the series of experiments and drafted the manuscript. SJB contributed significantly to the study design and planning of the series of experiments. HHvH provided critical intellectual input and interpretation. AH conducted the experiments and drafted portions of the manuscript. All authors contributed to critical revision toward and approved the final manuscript.

## Pre-publication history

The pre-publication history for this paper can be accessed here:

http://www.biomedcentral.com/2050-6511/15/58/prepub

## Supplementary Material

Additional file 1 **Results of preliminary experiments (Pilot studies 1 – 4).** Brief description of methodology and graphical depiction of results. Click here for file
