# Supplementary material for: A protocol for the delivery of cannabidiol (CBD) and combined CBD and ∆9-tetrahydrocannabinol (THC) by vaporisation
Source: BMC Pharmacol Toxicol. 2014 Oct 16;15:58. doi: 10.1186/2050-6511-15-58 (PMC4274767; doi:10.1186/2050-6511-15-58)

A protocol for the delivery of cannabidiol (CBD) and combined CBD and Δ^9-tetrahydrocannabinol (THC) by vaporisation

Solowij, Broyd, van Hell & Hazekamp

Additional file 1

Results of Preliminary Experiments

Pilot Study 1

• 100 mg crystalline CBD loaded in filling chamber on top of liquid pad

• Vaporisation at two different temperature settings: 210°C and 230°C

• For each temperature: three standard 60 cm balloons filled consecutively (labeled 210-1, 210-2, 210-3 and 230-1, 230-2, 230-3 in the figure below), vapour analysed by standard methods as described in the full paper

• Liquid pad was extracted to determine residual CBD (labeled 210-pad and 230-pad in the figure below)

• 'Missing' CBD can be accounted for by residue sticking to the inside of the filling chamber. This was clearly visible and could be felt as a sticky residue by touching it.

• The results indicate that 230°C is a better temperature for vaporising CBD to deliver a greater amount into the balloon, with less residue.

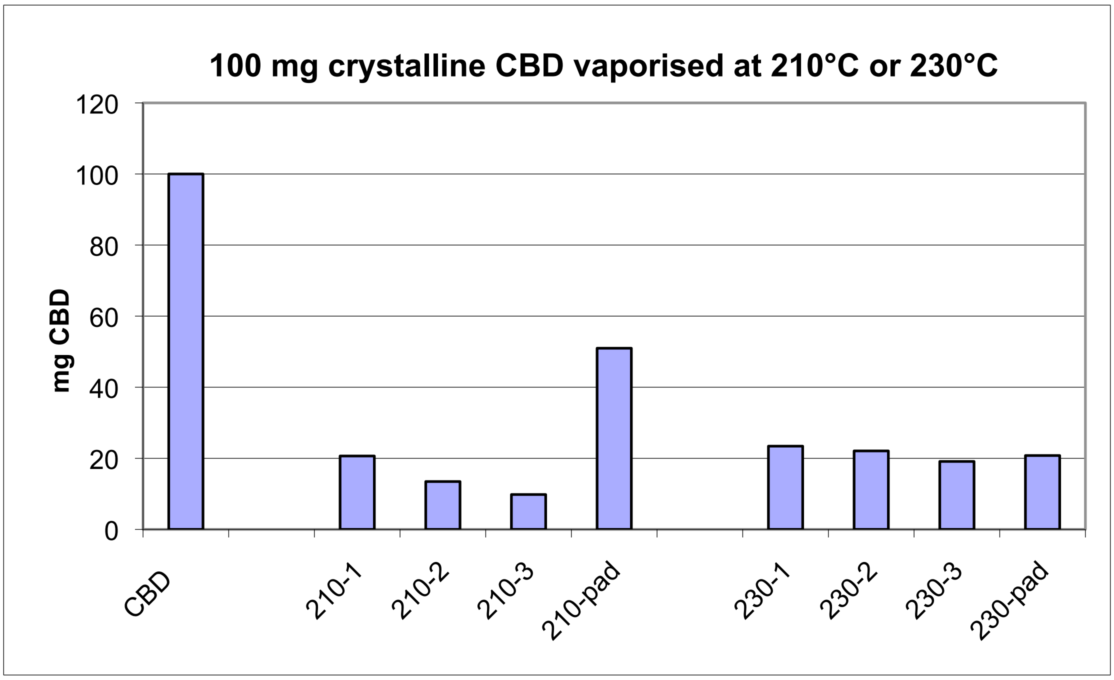

Pilot Study 2

- Temperature setting: 230°C
- Loading chamber filled with 100mg, 200mg and 300mg crystalline CBD on top of liquid pad
- Vaporisation into standard size balloon
- Vapour analysed for CBD content
- Results indicate that gains were made by loading 200mg CBD over 100 mg CBD in terms of vaporised dose delivered, but no further gains were made by loading 300mg CBD, and vaporisation was impeded by significant sticky residue clogging the liquid pad and chamber.
- This determined 200mg to be the optimal dose for most efficient vaporisation.

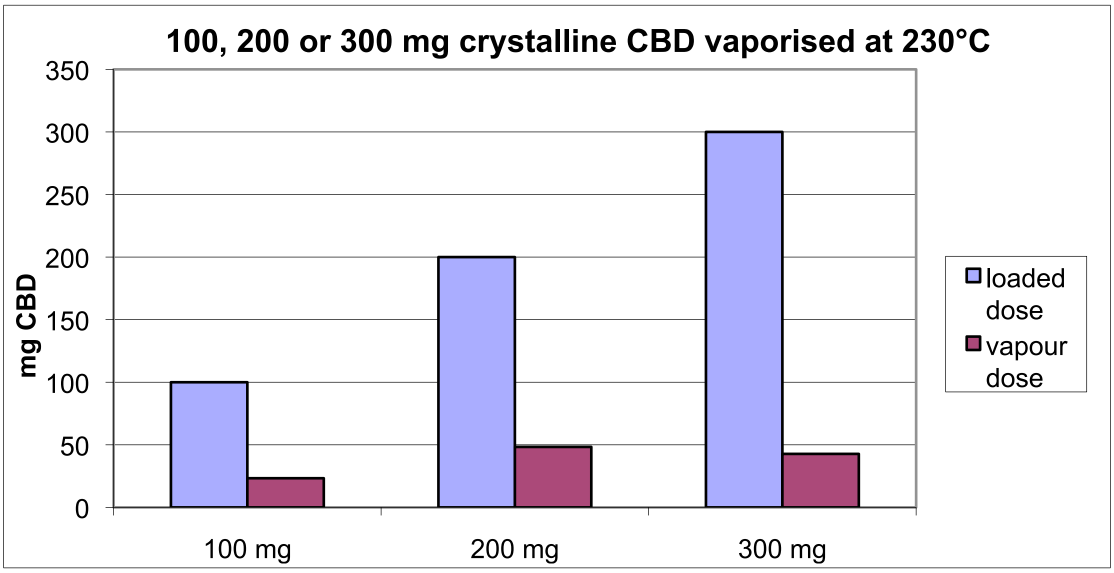

Pilot Study 3

• Temperature setting: 230°C

• Loading chamber filled with 200 mg crystalline CBD

• Compare vaporisation into normal balloon volume (60 cm) vs. XL balloon volume (90 cm)

• Vapour analysed for CBD content

• Results indicate that almost 25% of the loaded CBD is delivered into the normal balloon, and almost 30% delivered into the XL balloon from the same dose of CBD loaded.

• A substantially greater quantity of vapours must be inhaled from an XL balloon (1.5 times larger than normal balloon) for only an incremental increase in dose delivered.

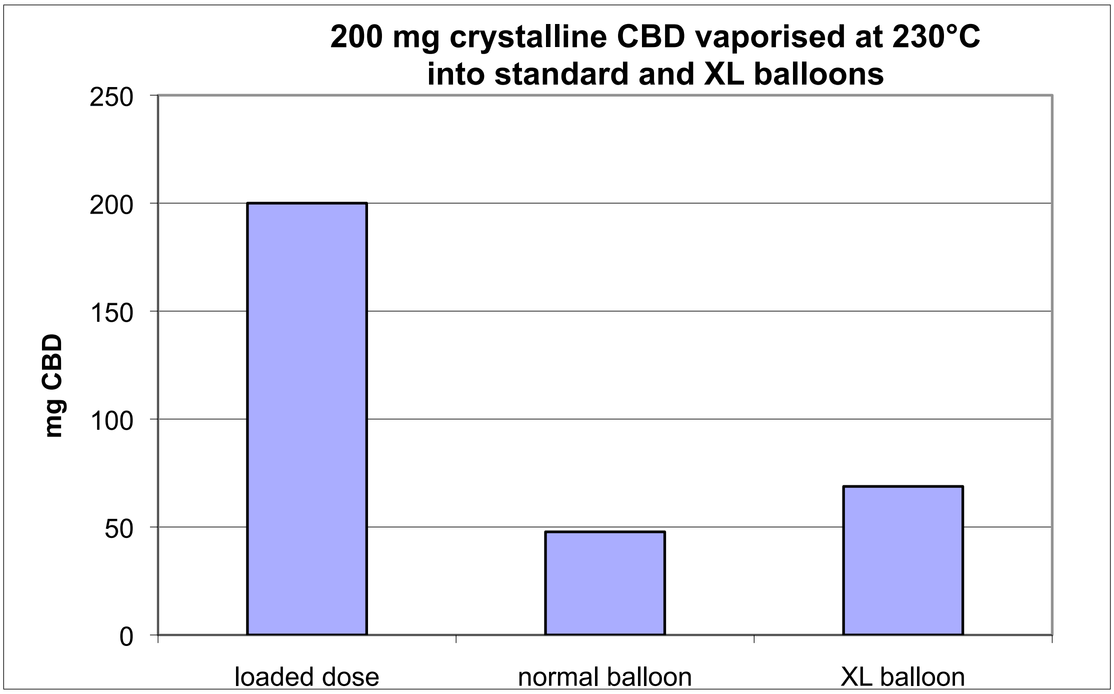

Pilot Study 4

• Temperature setting: 230°C

• Loading chamber filled with 100mg CBD and/or 10mg THC each in ethanolic solution

• Vapour analysed for CBD and THC content

• Results indicate no difference in vaporisation efficiency between CBD loaded in crystalline form (100 mg in Pilot Studies 1 and 2) or ethanolic solution (100mg here) and replicate reasonably well the findings of between 20% and 25% of the CBD loaded being delivered into the normal balloon (for 100mg or 200 mg doses). The addition of THC to the CBD in the loading chamber did not substantially alter the delivered dose of CBD. The dose of THC delivered was approximately 50%, thus decreasing by about half in the presence of CBD. This is likely due to a saturation effect occurring within the vapour.

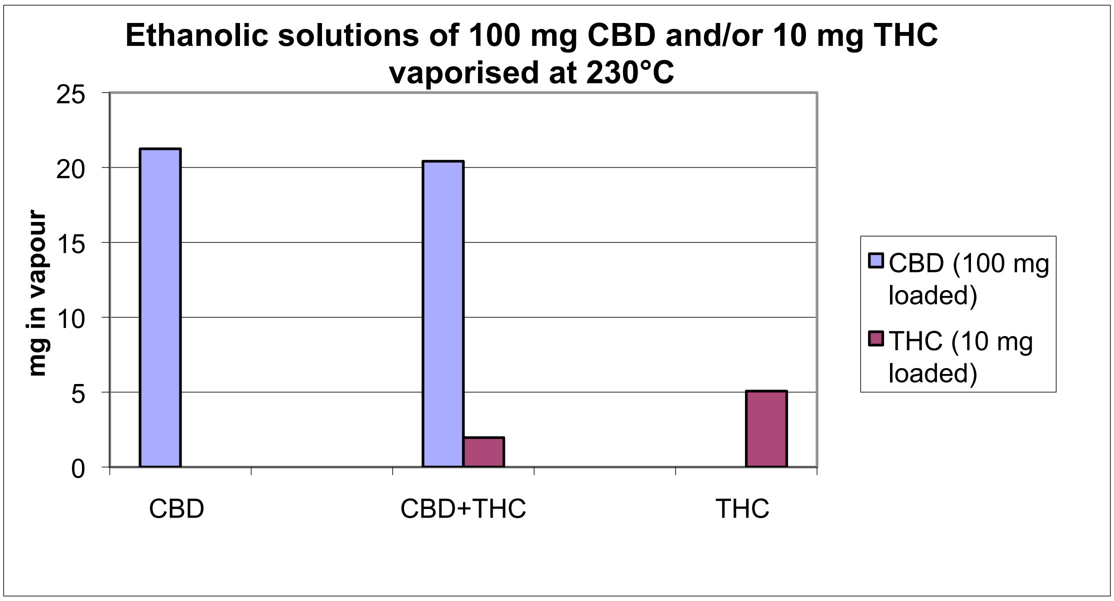

Supplement: Additional file 1 — Results of preliminary experiments (Pilot studies 1 – 4). Brief description of methodology and graphical depiction of results. [file 2050-6511-15-58-S1.pdf]
